# Dorsal Ganglion Cyst Excision Complicated by First Dorsal Metacarpal Artery Pseudoaneurysm: A Case Report

**DOI:** 10.7759/cureus.92788

**Published:** 2025-09-20

**Authors:** William Debrock, Pragna N Shetty, Grace A Longfellow, Gregory M Knoll

**Affiliations:** 1 Division of Plastic and Reconstructive Surgery, University of North Carolina at Chapel Hill School of Medicine, Chapel Hill, USA; 2 School of Medicine, University of North Carolina at Chapel Hill School of Medicine, Chapel Hill, USA; 3 Department of Orthopaedics, University of North Carolina at Chapel Hill School of Medicine, Chapel Hill, USA

**Keywords:** bleeding disorder, first dorsal metacarpal artery, ganglion cyst, pseudoaneurysm, wrist surgery

## Abstract

Ganglion cysts are the most common masses of the hand and wrist. With less invasive measures, these masses often recur, pointing to surgical excision as definitive treatment. Surgical excision of ganglion cysts is considered a safe surgery with a lower recurrence rate than other treatments. We present a rare case of a first dorsal metacarpal artery pseudoaneurysm after a dorsal wrist ganglion cyst excision in a patient with May-Hegglin anomaly. This report describes the presentation, surgical approach, and perioperative considerations in a patient with a hematologic condition.

## Introduction

Ganglion cysts are the most prevalent masses of the hand and wrist. These masses are mucin-filled sacks whose origin is from the joint space or tendon sheath [1,2]. Dorsal ganglion cysts are far more common than volar ganglion cysts and avoid involvement of the radial artery [2,3]. Despite the multifactorial etiology of ganglion cysts, management is straightforward. Surgical excision is considered definitive treatment. The most likely complication of surgery is recurrence, with arterial injury being low risk [2,4].

Pseudoaneurysms differ from aneurysms in that pseudoaneurysms are not contained by a layer of the vascular wall. Rather, they involve injury to the vessel with a sac, formed by coagulant products, encasing the hematoma [5]. Largely associated with the femoral artery, pseudoaneurysms are the most common iatrogenic complication from endovascular access needed for cardiac procedures [6]. These complications are rare in the upper extremity surgical population [7]. There are a few case reports describing pseudoaneurysms involved with volar ganglion cyst excisions and, to the authors’ knowledge, there are none concerning dorsal ganglion cyst excisions [8,9].

## Case presentation

A 35-year-old female presented for management of a left dorsal wrist ganglion cyst. The cyst had been present for over 20 years. Originally, the cyst ruptured soon after the patient noticed it; however, she required formal aspiration approximately 10 years later. Following aspiration, the cyst again recurred, after which she presented to our clinic for definitive management with surgical excision. On physical exam, the patient had a 1-cm, soft, mobile mass with transillumination over the dorsal scapholunate ligament consistent with a dorsal ganglion cyst. After a thorough discussion with the patient concerning the risks and benefits of surgery, the patient elected to proceed with the surgical excision of the ganglion. Of note, her medical history was significant for May-Hegglin Anomaly, a rare bleeding disorder caused by increased bleeding time and abnormal platelets [10]. Preoperative complete blood count panel was normal. She consulted with her hematologist for perioperative bleeding management. Her hematologist recommended preoperative tranexamic acid and desmopressin, along with postoperative tranexamic acid for five days. The patient was advised to avoid postoperative use of non-steroidal anti-inflammatory drugs or aspirin. 

Surgery was performed two months later using a forearm tourniquet and under local anesthetic with sedation. The incision was made over the dorsal radial wrist ganglion. The cyst was identified and traced to its exit point from the dorsal wrist capsule. The cyst and its stalk were excised en bloc from the dorsal wrist capsule, including a small window of the capsule itself. No dorsal capsule repair was required. After cauterization and irrigation of the excisional site, the wound was closed. The patient was placed in a plaster short arm splint, and the tourniquet was released with restoration of blood flow to the hand following placement of dressings. 

**Figure 1:**
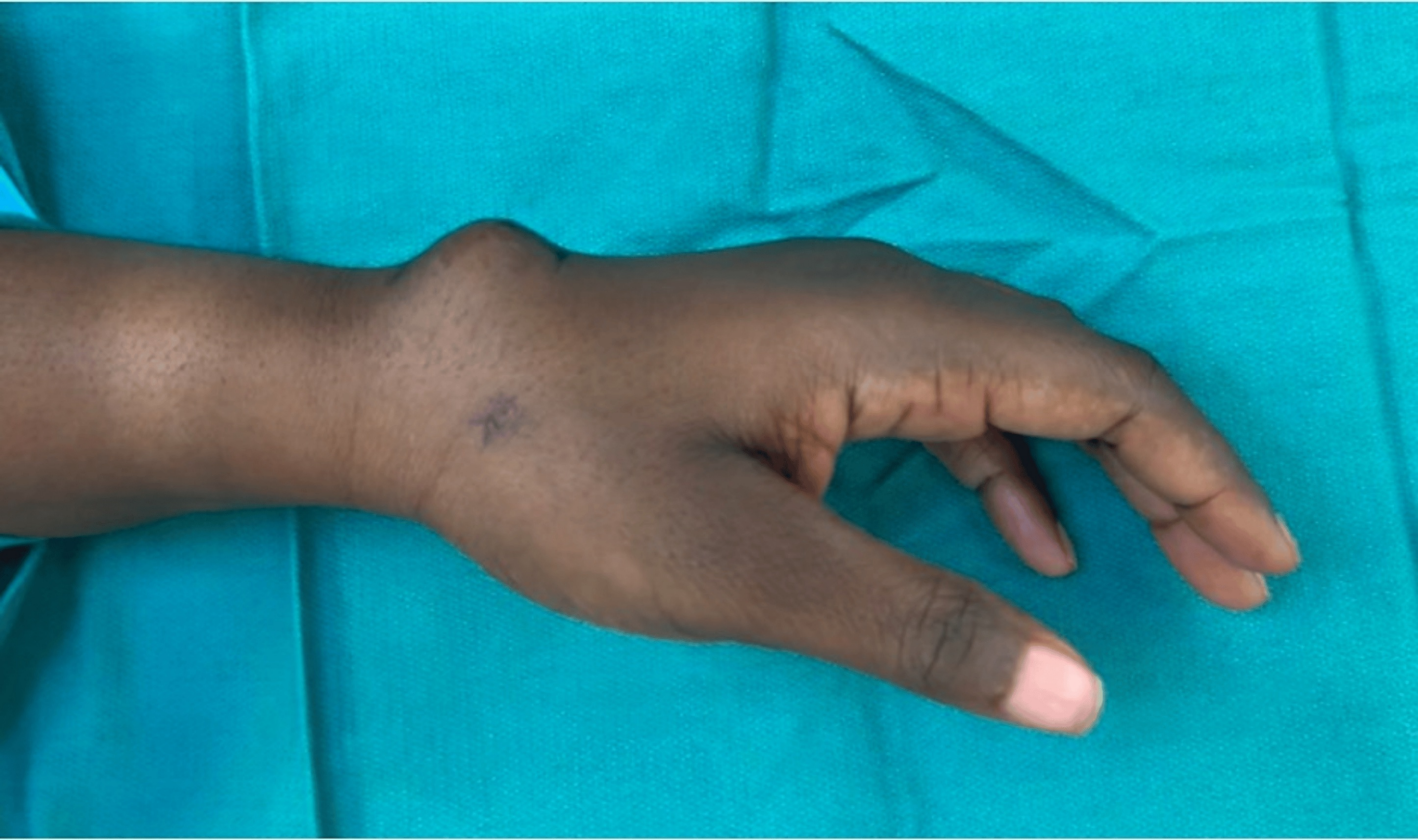
Preoperative photograph of the dorsal wrist showing a soft tissue swelling beneath the prior surgical incision.

**Figure 2:**
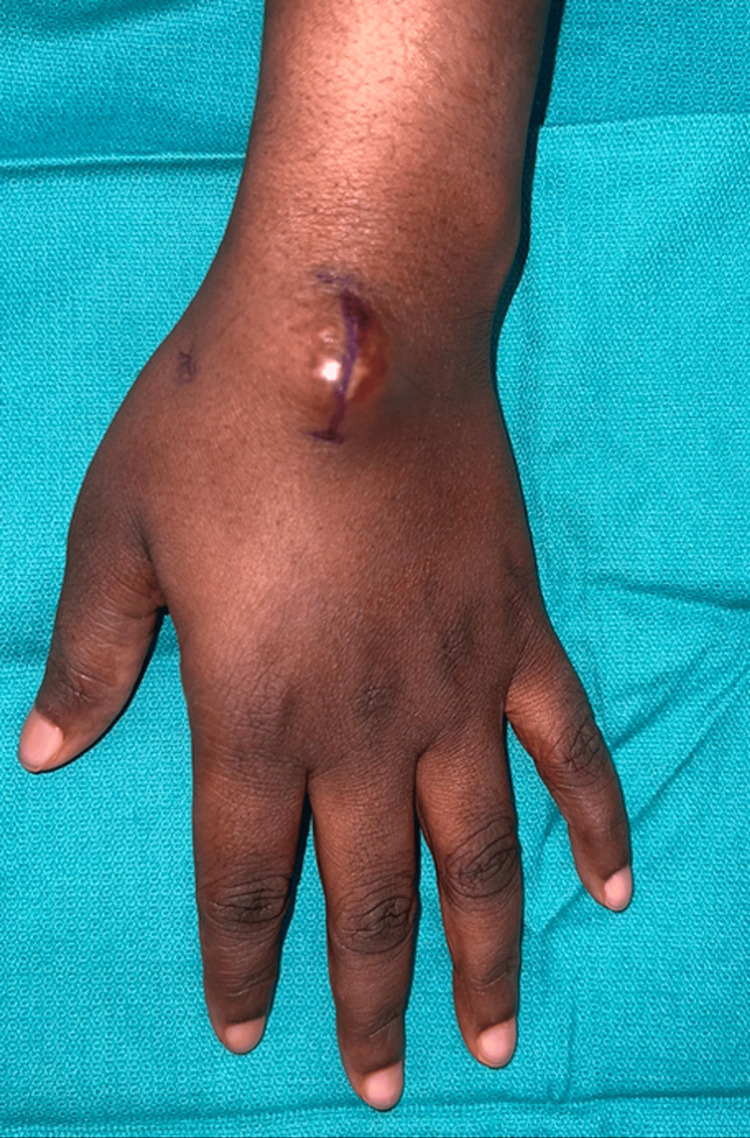
Close-up image and surgical marking of the dorsal wrist demonstrating a pseudoaneurysm beneath the skin.

The patient presented for a follow-up visit two weeks later. Her primary complaint was swelling over the surgical site. On exam, the patient had a small fluid collection directly underneath her incision. The patient was placed in a compression dressing and scheduled a follow-up visit in one month. She returned in one month with persistent swelling. On exam, there was an obvious pulsating mass underneath the surgical incision, giving concern for the presence of a pseudoaneurysm (Figure 1 and Figure 2).

The complication was discussed with the patient, and next-day surgery was scheduled.

**Figure 3:**
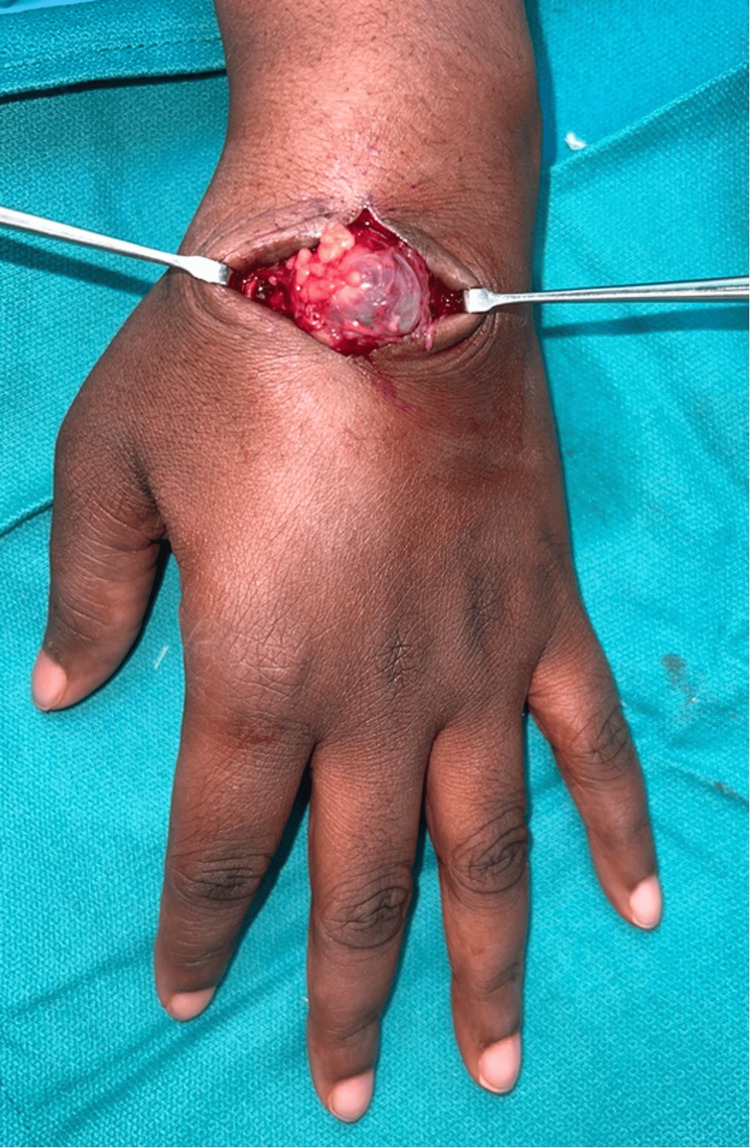
Intraoperative exposure revealing a vascular mass at the previous surgical site consistent with a pseudoaneurysm.

**Figure 4:**
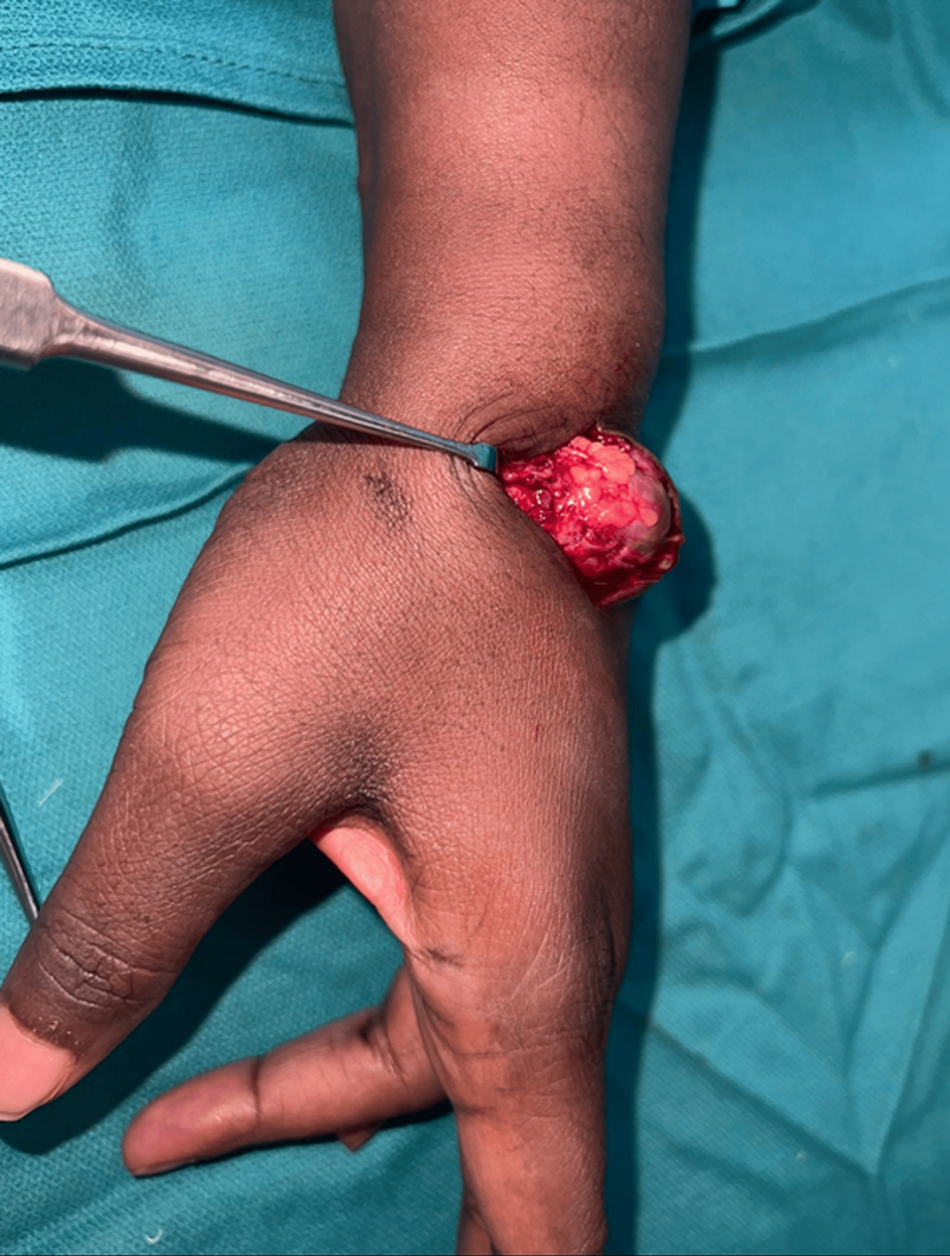
Operative view of the pseudoaneurysm.

In the operating room, the procedure was performed under local anesthetic with sedation and a forearm tourniquet. The healed surgical scar was incised, and the large vascular lesion was identified (Figure 3 and Figure 4).

**Figure 5:**
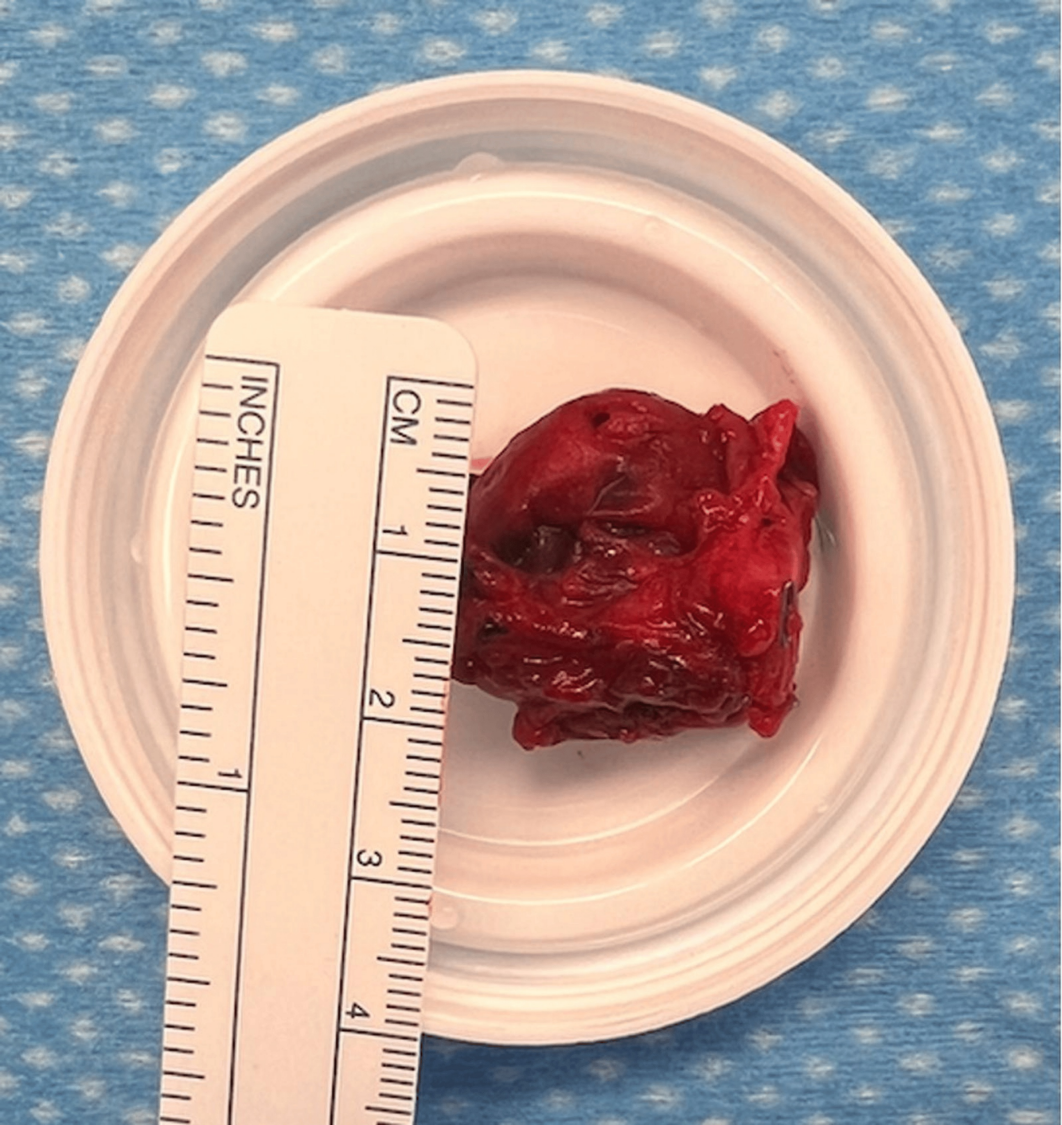
Gross surgical specimen of the pseudoaneurysm after excision.

The tourniquet was deflated to identify all feeding vessels to the pseudoaneurysm. The proximal feeding vessel was the first dorsal metacarpal artery. The vessel was traced to its branching point from the radial artery and ligated at the origin. The distal exiting vessel was also ligated. The vascular mass was sent for pathology. It was measured at a diameter of 2 cm (Figure 5). 

After thorough hemostasis, the wound was irrigated and closed. The patient was placed in a soft dressing. Her hematologist was once again consulted for perioperative bleeding management and recommended the same previous regimen.

**Figure 6:**
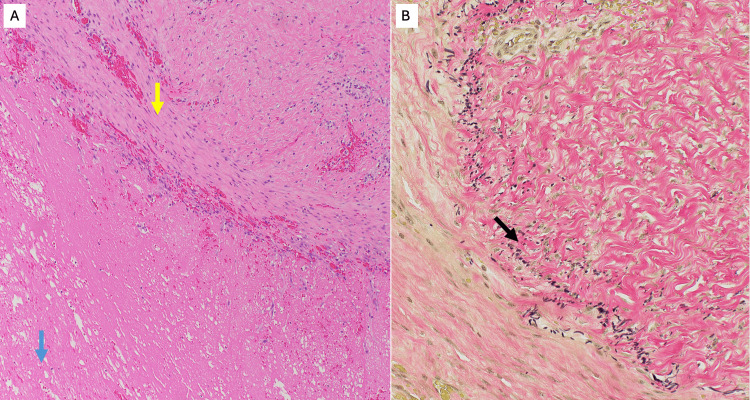
Photomicrographs of pseudodaneurysm with blood clot. (A) Hematoxylin and eosin (H&E)-stained section shows the wall of the pseudoaneurysm. A yellow arrow indicates the vascular intima; a blue arrow highlights the associated blood clot. (B) Elastic tissue stain demonstrates discontinuous fragments of the internal elastic lamina (black arrow), consistent with a pseudoaneurysm, as not all arterial wall layers are involved in the dilatation.

The patient presented for a follow-up visit one week later. Pathology confirmed the surgical specimen was a pseudoaneurysm (Figure 6).

On exam, her incision was healing appropriately with no concerning swelling, drainage, or erythema. Her sutures were removed, and she was cleared for light activity. She had a final follow-up visit one month later without any concerns on exam.

## Discussion

Ganglion cysts are the most common masses of the hand and are definitively managed with surgical excision [1-3]. Approximately 70% of ganglion cysts are dorsal rather than volar. The primary complication with open excision is recurrence, which is highest, up to 20%, with volar ganglions [11,12]. Additionally, volar ganglions can involve the radial artery. Open excision for volar ganglions necessitates precise dissection and preservation of the radial artery while completely removing the cyst wall [3]. Incidence of radial artery injury in these cases is between 1% and 16% [13]. There are a few case reports describing pseudoaneurysm of the radial artery as a complication following volar wrist ganglion excision [8,9]. The first of these reports was in 1996. The authors describe the diagnosis of the complication with ultrasound and treatment with excision. They recommend deflating the tourniquet prior to closure in future cases to prevent the same complication [8]. Another paper reports radial artery pseudoaneurysm formation after arthroscopic resection in a patient with hemophilia. Diagnosis was made by physical exam on postoperative day 3, and the patient was treated with resection [9]. Our case is the first, to the authors’ knowledge, which diagnosed and managed a first dorsal metacarpal artery pseudoaneurysm after dorsal ganglion cyst excision. 

While rare in the upper extremity, pseudoaneurysms are associated with lower extremity endovascular access as an iatrogenic complication [6,7]. Typically, after a thorough physical exam, duplex ultrasonography is the preferred imaging for diagnosis [14,15]. For our patient, her clinical exam at one month was suggestive of a pseudoaneurysm and did not require an ultrasound for confirmation. However, at her one-week follow-up visit, when the diagnosis remained unclear, an early Doppler ultrasound could have differentiated a postoperative seroma or hematoma from a vascular lesion. Early use of Doppler ultrasound in such scenarios is a valuable diagnostic step and may expedite recognition of vascular complications before they become clinically apparent. Habib et al. demonstrated that ultrasound with color Doppler is effective in classifying superficial soft-tissue vascular anomalies and in excluding non-vascular lesions, supporting its role in early postoperative evaluation [16].

Management options depend on the features of the pseudoaneurysm. If there are concerning signs such as an expanding hematoma, hemodynamic instability, or necrotic tissue, then emergent surgical management is needed. For non-emergent situations, treatment options include observation with spontaneous thrombosis, ultrasound-guided thrombin injection, and surgical excision [6,15,17-19]. Clearly, our patient failed observation, which indicated surgical excision. 

Our patient was unique in that she had had a diagnosis of May-Hegglin anomaly, a rare bleeding disorder [10]. This, in combination with her obesity and female sex, likely put her at an increased risk for pseudoaneurysm [20]. Despite perioperative recommendations from her hematologist, the arterial injury during the procedure resulted in a pseudoaneurysm. Similar to the original case report in 1996, we believe deflation of the tourniquet prior to closure would have allowed for identification of the injury [8]. Furthermore, it may have been appropriate for ultrasound evaluation at the patient’s first follow-up visit for prompt diagnosis of the vascular mass. We recommend consideration of early tourniquet deflation for arterial injury rule out for patients undergoing ganglion cyst excision with risk factors for pseudoaneurysm formation.

## Conclusions

First dorsal metacarpal artery pseudoaneurysm formation following excision of a dorsal ganglion cyst is extremely rare, and to our knowledge, this case is among the first to report the complication. The development of a new, pulsatile mass in the postoperative period should raise suspicion for a pseudoaneurysm, especially in patients with risk factors such as obesity, female sex, or a bleeding disorder. While the diagnosis in our patient was clinically apparent, this case underscores the importance of ultrasound early in the postoperative course when a new mass is present but the diagnosis is uncertain. Early imaging can distinguish benign fluid collections from vascular lesions, allowing for timely and appropriate intervention. Management ranges from observation (e.g., compressive dressing) to surgical intervention and should be guided by the presence of concerning features such as an expanding hematoma, hemodynamic instability, or necrotic tissue, which necessitate emergent surgical excision. This case also highlights the importance of intraoperative preventative steps, such as deflating the tourniquet prior to closure to assess for unrecognized arterial injury. We recommend that surgeons consider this step in anatomically complex regions or in patients at increased risk for vascular complications to reduce the likelihood of delayed diagnosis and reoperation.
